# Case report: Clinical complete response in advanced ALK-positive lung squamous cell carcinoma: a case study of successful anti-PD-1 immunotherapy post ALK-TKIs failure

**DOI:** 10.3389/fimmu.2024.1360671

**Published:** 2024-02-06

**Authors:** Chen Yang, Rui Zeng, Yawen Zha, Yani Li, Ting Wang, Ruolan Zhao, Minying Li, Jingjing Zhang

**Affiliations:** ^1^ Zhongshan City People’s Hospital, Xinxiang Medical University, Xinxiang, China; ^2^ Department of Radiotherapy, Zhongshan City People’s Hospital, Zhongshan, China; ^3^ Department of Imaging, Zhongshan City People’s Hospital, Zhongshan, China

**Keywords:** ALK-positive, lung squamous cell carcinoma, clinical complete response, ALK-TKI, immunotherapy

## Abstract

In patients with advanced lung adenocarcinoma (LADC) harboring the echinoderm microtubule-associated protein-like 4 (EML4) -anaplastic lymphoma kinase (ALK) rearrangement, targeted therapy typically demonstrates superior efficacy as an initial treatment compared to chemotherapy. Following resistance to ALK-tyrosine kinase inhibitors (TKIs), regimens incorporating platinum-based dual agents or combined with bevacizumab often show effectiveness. However, therapeutic alternatives become constrained after resistance develops to both TKIs and platinum-based therapies. Given that the majority of ALK-positive non-small cell lung carcinomas (NSCLC) are LADC, the benefits of TKIs for patients with ALK-positive lung squamous cell carcinoma (LSCC) and the optimal treatment strategy for these patients remain a subject of debate. In this case study, we report on a patient with advanced LSCC, in whom the EML4-ALK rearrangement was identified via ARMS-PCR (Amplification Refractory Mutation System-Polymerase Chain Reaction). The patient underwent oral treatment with crizotinib and alectinib, showing effectiveness in both first-line and second-line ALK-TKI therapies, albeit with limited progression-free survival (PFS). Subsequent resistance to second-generation TKI was followed by the detection of tumors in the left neck region via computed tomography (CT). Biopsy pathology revealed non-squamous cell carcinoma, and subsequent treatment with platinum-based double-drug therapy proved ineffective. Further analysis through next-generation sequencing (NGS) indicated ALK negativity but a high expression of programmed death-ligand 1 (PD-L1). Immunotherapy was then initiated, resulting in a PFS of over 29 months and clinical complete remission (cCR). This case underscores the potential benefit of ALK-TKIs in patients with ALK-positive LSCC. Resistance to second-generation TKIs may lead to ALK negativity and histological transformation, highlighting the necessity of repeated biopsies post-TKI resistance for informed treatment decision-making. As of November 2023, imaging studies continue to indicate cCR in the patient, with a survival time exceeding 47 months.

## 1 Introduction

Lung cancer remains the foremost oncological challenge globally (1). Within this domain, Non-Small Cell Lung Cancer (NSCLC) is the predominant subtype, comprising approximately 85% of all lung cancer cases (2). NSCLC primarily manifests in two pathological forms: Lung Squamous Cell Carcinoma (LSCC) and Lung Adenocarcinoma (LADC). Recent advancements in the understanding of NSCLC have ushered in an era of molecularly-tailored treatments, particularly focusing on driver genes, which are pivotal in tumor developments (3, 4). Targeted therapies, designed to address specific driver gene mutations, have shown promise in enhancing the Objective Response Rate (ORR) and Progression-Free Survival (PFS) in patients, thereby augmenting overall quality of life and extending Overall Survival (OS) (3, 4). One significant driver gene in NSCLC is the Anaplastic Lymphoma Kinase (ALK) fusion gene, identified in about 5% of NSCLC patients, predominantly within the LADC subgroup (5). Conversely, the occurrence of ALK fusion genes in LSCC is considerably rare (6). Consequently, the effectiveness of ALK-Tyrosine Kinase Inhibitors (ALK-TKIs) in LSCC patients remains less clear, due to their low representation in clinical studies. This paper discusses a case study of a patient with advanced LSCC, exhibiting the echinoderm microtubule-associated protein-like 4 (EML4) - ALK rearrangement, who responded favorably to anti-programmed death 1 (PD-1) immunotherapy post ALK-TKIs failure.

## 2 Case presentation

**Figure 1:**
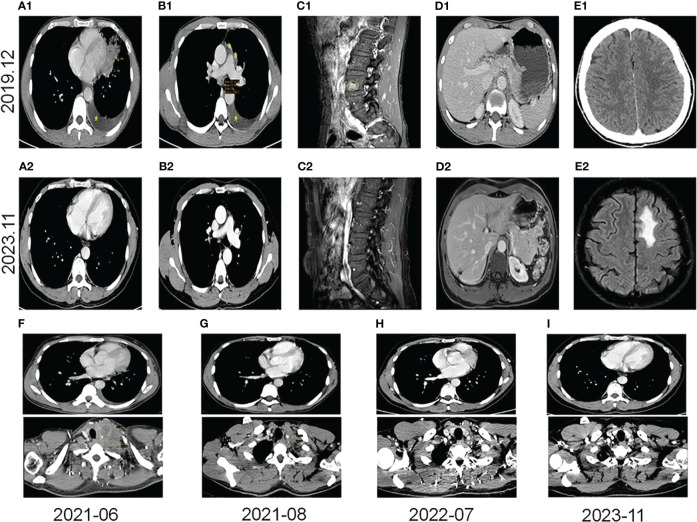
Images from pre-targeted therapy (December 2019). **(A1)** Soft tissue mass near the heart margin of the lower lingual segment of the left upper lobe (x) and a small effusion of the left thoracic cavity (*) on the CT scan. **(B1)** Lymph node metastases (○), pleural metastases (#), and a small effusion of the left thoracic cavity (*) on the CT scan. **(C1)** Spinal metastasis on the MRI scan (○). **(D1)** A CT scan of liver. **(E1)** A CT scan of the brain. Images from post-immunotherapy (November 2023). **(A2)** No mass at the left superior lobe lingual segment on the CT scan. **(B2)** No nodules of mediastinum and hilum on the CT scan. **(C2)** Inactive of the spinal metastasis on the MRI scan. **(D2)** A change after ablation of the liver S7 segment on the MRI scan. (**E2)** Radionecrotic inflammatory response of the left frontal lobe on the MRI scan. Changes in the left lung mass and the left supraclavicular fossa lymph nodes on the CT scans during immunotherapy **(F–I)**. **(F)** The left supraclavicular fossa mass (52x69mm) and the left lung mass before immunotherapy; **(G)** The left supraclavicular fossa mass (19x20mm) and on the left lung mass after 2 months of immunotherapy. **(H)** Without any masses after 13 months of immunotherapy. **(I)** Without any masses after 29 months of immunotherapy.

**Figure 2:**
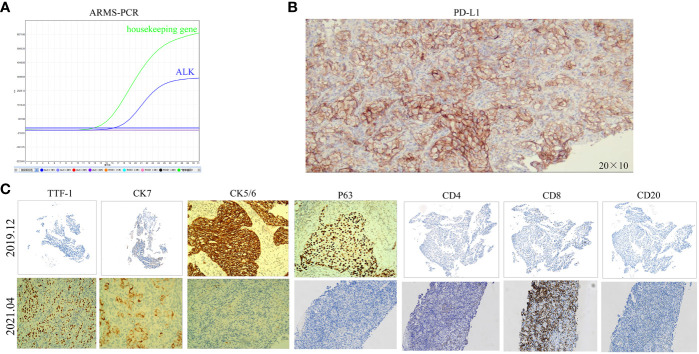
The ARMS-PCR result of the ALK gene in December 2019 and the immunohistochemistry results before and after resistance to ALK-TKIs. **(A)** The ARMS-PCR shows ALK fusion in December 2019. **(B)** PD-L1 (IHC)-TPS of 90% (200x)after resistance to ALK-TKIs. **(C)** CK5/6(100x), P63(100x), and CK7(100x)-positive in December 2019; CD8(100x)-scattered in a few positive in December 2019, about 10% of lymphocytes; CD4(100x), CD20(100x), and TTF-1(100x)-negative in December 2019; CK7(200x), TTF-1(100x), CK5/6(200x), and CD8(200x)-positive in April 2021; P63(200x), CD4(200x), and CD20(200x)-negative in April 2021.

A 40-year-old male patient, with no history of smoking or drinking, presented to our clinic in December 2019 with complaints of a mild dry cough persisting for over two months and slight left chest pain lasting for more than a month. A chest computed tomography (CT) scan revealed the presence of a 42x21mm mass located centrally in the left lung, accompanied by enlarged ipsilateral hilar and mediastinal lymph nodes, an ipsilateral pleural tubercle, and a small pleural effusion (**Figures 1A1, B1**). Further evaluation through a magnetic resonance imaging (MRI) scan indicated the presence of vertebral metastases (**Figure 1C1**). No metastatic lesion was observed in the liver (**Figure 1D1**) or brain (**Figure 1E1**). Pathological examination of the mass in the left lung confirmed the diagnosis of poorly differentiated LSCC. Immunohistochemical analysis revealed positive staining for CK, CK7, CKHMW, CK5/6, P63, and CK7, with a Ki-67 proliferation index of 40% (staining for CK5/6, P63 and CK7 in **Figure 2C**). However, chromogranin A, Syn, CD56, NapsinA, CD4, CD8, CD20 and thyroid transcription factor-1 (TTF-1) showed negative staining(staining for CD4, CD8, CD20 and TTF-1 in **Figure 2C**). An EML4-ALK fusion was detected using ARMS-PCR (Amplification Refractory Mutation System-Polymerase Chain Reaction) analysis(**Figure 2A**). The pathology of the left pleural effusion indicated the presence of suspected tumor cells. The patient had a Eastern Cooperative Oncology Group Performance Status 1 (ECOG PS) and a squamous cell carcinoma antigen (SCCA) level of 3.2 ng/ml (normal range: 0.6-2.3 ng/ml).

**Figure 3:**
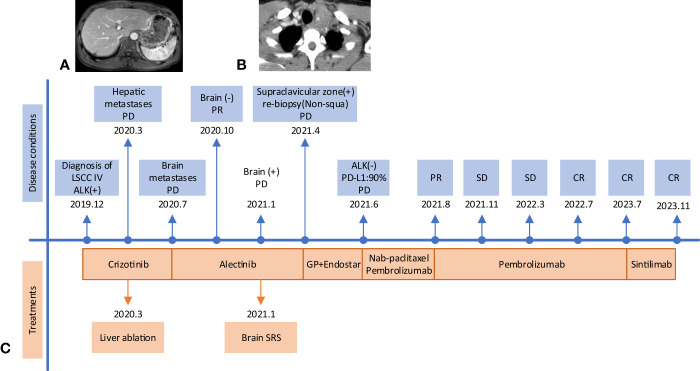
The timeline treatment and some progressive images. **(A)** Liver S7 nodule (11mm×10mm) on an MRI scan in March 2020. **(B)** A mass (32×47mm) fused by lymph nodes in the left supraclavicular fossa on the CT scan in April 2021. **(C)** The treatment timeline. LSCC, lung squamous cell carcinoma; PD, progressive disease; PR, partial response; SD, stable disease; CR, complete response; SRS, Stereotactic radiosurgery.

**Figure 4:**
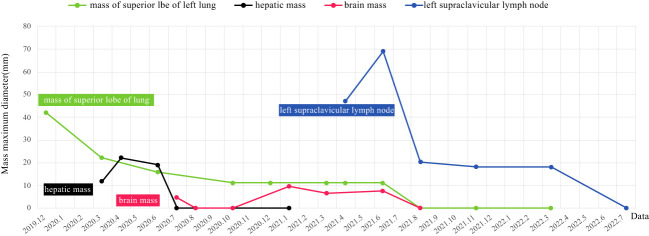
Imaging changes of the left lung, liver, brain, and left supraclavicular fossa mass during treatment in our case(the black and red lines on the horizontal coordinate indicate that inactivity of the metastases on the MRI scans).

The patient was diagnosed with stage IVB ALK-positive advanced LSCC (cT2bN2M1c). Subsequently,the patient was prescribed crizotinib (250mg bid) in December 2019. A CT scan in March 2020 revealed a reduction in the size of the left mass (22x13mm), mediastinal and left hilar lymph nodes, and pleural tubercle. Concurrently, an MRI scan detected a hepatic S7 nodule, suspected to be a metastasis (11mm×10mm), as shown in **Figure 3A**. According to the Response Evaluation Criteria in Solid Tumors (RECIST) version 1.1, the condition was assessed as progressive disease (PD). He still had a PS of 1. In March 2020, the patient underwent radiofrequency and chemical ablation for the liver metastasis while continuing crizotinib. By July 2020, MRI scans demonstrated post-treatment alterations in the liver S7 metastasis, including coagulation and necrosis. However, a new T2W-FLAIR hypersignal focus, approximately 4.6mm in diameter, was identified in the left frontal lobe, suggesting potential brain metastasis. This again was evaluated as PD. The patient had a PS of 1 at the time. Consequently, the treatment regimen was switched to alectinib (600mg bid) in July 2020. Subsequent brain MRIs in August and October 2020 showed no evidence of brain metastasis. However, in January 2021, the patient experienced intermittent dizziness with PS 1, and an MRI revealed a nodule in the left frontal lobe with surrounding brain tissue edema, indicative of metastasis (9x9.5mm). Stereotactic radiosurgery (SRS) for the brain metastasis was performed from February 3 to February 7, 2021 (30Gy in 3Gy fractions), while maintaining the alectinib regimen. This treatment led to an improvement in the patient’s dizziness. A follow-up brain MRI in August 2021 showed changes in the left frontal mass consistent with post-radiotherapy effects for brain metastasis. Periodic subsequent evaluations have indicated no active tumor in the left frontal lobe lesion and no new brain metastases. June and October 2020 CT scans indicated a reduction in the size of the left lung mass. Subsequent regular CT scans from October 2020 to June 2021 suggested that the size of this mass remained largely unchanged. However, in April 2021, the patient noted a mass on the left side of his neck with PS 2, accompanied by symptoms, such as severe hoarseness and moderate dizziness. A CT scan at this time revealed no significant change in the left lung mass size, but there was an apparent fusion of lymph nodes in the left supraclavicular fossa into a larger mass measuring 32×47mm, as depicted in **Figure 3B**. This observation led to the suspicion of disease progression. A puncture biopsy of the left supraclavicular lymph node was conducted. Pathological examination identified the presence of metastatic poorly differentiated carcinoma, likely originating from the lung. Immunohistochemical analysis demonstrated that the tumor cells were positive for cytokeratin (CK), CK7, TTF-1, CK5/6, CD8 and Ki-67 (60%) (staining for CK7, TTF-1, CK5/6 and CD8 in **Figure 2C**), while negative for NapsinA, P40, P63, PAX-8, CD10, CgA, CD4, CD20, CD56, Hepatocyte, TG, and BEER(staining for P63, CD4, and CD20 in **Figure 2C**). SCCA levels were within normal ranges. On May 1 and May 22, 2021, the patient received GP chemotherapy (gemcitabine 1.6g on days 1 and 8, and Nedaplatin 40mg on days 1-3) along with recombinant human endostatin (Endostar, 210mg). During the period of chemotherapy session, the patient showed second-degree vomiting and diarrhea, but no adverse hematological reactions were observed. A follow-up CT scan in June 2021 (**Figure 1F**) revealed an increase in the size of both the bilateral hilar and left supraclavicular fossa lymph nodes, measuring 52x69mm, indicating a progression of the metastatic disease. The disease was evaluated as PD; the patient had a PS score of 3, along with persistent hoarseness and dizziness. To further investigate, left cervical lymph node and peripheral blood samples underwent next-generation sequencing (NGS) of 550 tumor-related genes. Under the BioProject ID PRJNA1062325, the raw sequencing date have been submitted to the Sequence Read Archive (SRA). The NGS findings indicated a negative status for ALK fusion, while programmed death-ligand 1 (PD-L1) expression was significantly positive (90%) (PD-L1 expression in **Figure 2B**). Subsequently, the patient received treatment with nab-paclitaxel (400mg, day 1) and the PD-1 monoclonal antibody Pembrolizumab (200mg, day 1) on June 18 and July 9, 2021. An August 2021 CT scan revealed a notable reduction in the size of the pulmonary nodules, which had nearly disappeared, and a decrease in the size of the left supraclavicular fossa nodule to 19x20mm, as shown in **Figure 1G**. However, after using nab-paclitaxel, the patient developed neurotoxicity and numbness in his hands and feet and did not experience hematologic toxicity. The patient opted against further chemotherapy, continuing treatment with Pembrolizumab until July 2023. The patient’s neurotoxic symptoms disappeared about three months after the withdrawal of nab-paclitaxel. Regular follow-ups from August 2021 to March 2022 classified the patient’s condition as stable disease (SD). A CT scan in July 2022 (**Figure 1H**) found no pulmonary or supraclavicular nodules. Periodic assessments from July 2022 to November 2023 confirmed the maintenance of no pulmonary or supraclavicular nodules. The symptom of severe hoarseness lasted about one year and then gradually improved. In August 2023, the patient had a PS of 0 and elected to switch to sintilimab (PD-1 monoclonal antibody, 200 mg), citing economic reasons after two years of pembrolizumab treatment without medicinal donations. As of November 2023, chest CT scans showed no detectable lesions (**Figures 1I**, **A2, B2**), and MRI scans revealed no active lesions in the spine, liver, and brain (**Figures 1C2, D2, E2**). With PS 0, the patient is satisfied with the therapeutic effect and the current quality of life. The timeline of the treatment process is depicted in **Figure 3C**. **Figure 4** illustrates the dynamic changes in lesions located in the left lung, liver, brain, and left supraclavicular fossa throughout the course of treatment. Informed consent was obtained from the patient for the purpose of this case report.

## 3 Discussion

In the realm of LADC and LSCC, the presence of ALK-positive cases is relatively rare, occurring in less than 5% and 1% of patients respectively (5–7). The efficacy of targeted therapy for ALK-positive LSCC patients remains uncertain. A study by Lewis et al. examined six LSCC patients with EML4-ALK rearrangement and reported a median PFS of 2.8 months (ranging from 1.8 to 6.3 months) and OS of 8.3 months (ranging from 3.2 to 32.1 months) when treated with ALK-TKIs as first or second-line therapy (8). Yuan et al. documented a case of advanced LSCC with ALK positivity treated with first-line alectinib, resulting in a PFS of 4.5 months and an OS of 6 months (9). Furthermore, Meng et al. presented a single-center case review of 31 ALK-positive LSCC patients, of whom 20 received ALK-TKIs as their initial or subsequent treatment. The median PFS in this group was 6.4 months ( ± 4.4 months), notably shorter than that observed in ALK-positive LADC patients treated with ALK inhibitors (10). Lu et al. reviewed 11 ALK-positive LSCC patients and noted that their PFS, whether treated with crizotinib or alectinib, was shorter compared to LADC patients. They highlighted a case where a patient with EML4-ALK (V1) fusion and a high TP53 co-mutation achieved the longest PFS of 19 months on ensartinib, before switching to loratinib due to disease progression and diminished effectiveness (11). In our study, the PFS of ALK-positive LSCC patients treated with first- or second-line ALK-TKI mirrored findings from previous literature (8–11), yet the survival time exceeded 47 months, surpassing all similar reports (9, 12). Currently, the patient maintains a performance status (PS) score of 0.

The management of brain metastases (BMS) presents a significant clinical challenge in ALK-positive NSCLC. Patients with ALK-positive NSCLC are more susceptible to BMS compared to those with epidermal growth factor receptor (EGFR)-positive NSCLC (12). Approximately 30% of these patients present with central nervous system (CNS) metastases at the time of initial diagnosis (13), and about 58% develop CNS metastases within three years (14). The first-line standard treatment for advanced ALK-positive NSCLC typically involves ALK-TKIs (14, 15). However, the first-generation ALK-TKIs demonstrate limited efficacy in controlling brain metastases due to poor penetration of the blood-brain barrier. In contrast, second-generation ALK-TKIs offer enhanced blood-brain barrier permeability, resulting in better management of brain metastases. Consequently, second or third-generation ALK-TKIs are now recommended as the first-line treatment for ALK-positive NSCLC patients without BMS to decrease the likelihood of developing BMS. BMS significantly impacts the quality of life and survival prognosis of patients. Advances in radiotherapy, such as stereotactic radiotherapy (SRT) and SRS, have improved the precision of brain radiotherapy for lung cancer patients with brain metastases. Local radiotherapy not only augments disease control but also increases the permeability of the blood-brain barrier (16). In our case study, a patient with advanced ALK-positive NSCLC developed brain metastasis seven months after initiating treatment with crizotinib. This metastasis resolved within a month following a switch to alectinib. However, the brain metastasis reemerged six months after starting the second-generation ALK-TKI. To manage this, SRS was employed alongside continued systemic therapy. As of November 2023, the metastasis in the left frontal lobe exhibited no tumor activity, and no new metastases were detected in the brain.

In nearly half of the ALK-positive NSCLC patients experiencing progression on second-generation ALK-TKIs, no ALK mutations are detected at the time of clinical relapse. This suggests the development of ALK-independent resistance, and such patients derive only limited benefit from subsequent-generation ALK-TKIs (17, 18). The transformation of a tumor into a different histologic subtype is often linked to a loss of dependence on the initial oncogenic driver, contributing to drug resistance (19). Although most newly diagnosed cases of ALK-positive NSCLC are LADC, transformations to small cell lung cancer (SCLC) have been observed in patients treated with all generations of ALK-TKIs. This occurrence, however, is infrequent, with a rate of less than 3% as determined by retrospective analyses (20–23). To date, there have only been six reported cases documenting such a transformation to SCLC in the context of ALK-positive lung cancer. Post-histological transformation, patients with ALK-positive NSCLC generally exhibit rapid disease progression, with both targeted therapy and chemotherapy proving ineffective in most cases (20, 24–28). Remarkably, there has been only one documented instance where a patient, transformed to SCLC following ALK-TKI treatment, responded positively to chemotherapy and immunotherapy (28). Yan et al. presented a case of ALK-rearrangement-positive adenocarcinoma with high expression of PD-L1 that was transformed into LSCC after administration of alectinib (29). Reports indicate an increased likelihood of NSCLC transforming into SCLC under the stress of targeted therapy (30). This transformation seems more prevalent following treatment with second-generation ALK-TKIs, possibly due to the heightened exposure stress experienced by tumor cells under these medications. To adapt to this stress, the cells may undergo biological or histological changes (31). Due to the low incidence of ALK-positive LSCC, no cases of histological transformation of ALK-positive LSCC have been reported. In the case presented, the patient exhibited elevated serum SCCA levels prior to targeted therapy, which normalized post-second-generation TKI treatment. The tumor’s pathology shifted from LSCC to non-squamous cell carcinoma, and no ALK mutations were detected through NGS. This could be indicative of an ALK-independent resistance mechanism, although tumor heterogeneity cannot be ruled out. Following resistance to the second-generation TKI, the disease progressed rapidly, and platinum-based chemotherapy was ineffective. However, due to high PD-L1 expression, the patient showed significant response to immunotherapy. The PFS with immunotherapy exceeded 29 months, and the patient achieved a clinical complete remission (cCR), with a survival time of over 47 months. This case suggests a novel approach for treating advanced ALK-positive LSCC. It again implies that histological transformation might occur following ALK-TKI resistance, underlining the importance of repeat biopsies for patients resistant to TKIs.

In this instance, while targeted therapy was initially effective, the PFS achieved was limited. Notably, post-targeted therapy, the patient’s tumor pathology transitioned from LSCC to non-squamous cell carcinoma. Additionally, NGS did not identify any gene mutations. The most likely explanation is that TKIs put more severe selective pressure on the squamous carcinoma cells during TKI-therapy. After overcoming the evolutionary process of selective pressure, TKI-resistant tumor cells undergo biological changes more frequently. These changes do not eliminate the possibilities of tumor heterogeneity, a factor that cannot be discounted. A significant improvement was observed after the patient was transitioned to immunotherapy. To the best of our knowledge, this is the only case reported in the literature about an ALK-positive advanced LSCC patient achieving cCR following treatment with ALK-TKIs, localized treatment, and immunotherapy. However, there are some limitations in this case, such as the lack of pathology of liver matastasis and PD-L1 expression level before ALK-TKIs therapy. As of now, the patient’s survival time has exceeded 47 months, underscoring the potential efficacy of this treatment approach.

## Data availability statement

The original contributions presented in the study are included in the article/supplementary materials, further inquiries can be directed to the corresponding author/s.

## Ethics statement

Ethical approval was not required for the study involving humans in accordance with the local legislation and institutional requirements. Written informed consent to participate in this study was not required from the participants or the participants’ legal guardians/next of kin in accordance with the national legislation and the institutional requirements. Written informed consent was obtained from the individual(s) for the publication of any potentially identifiable images or data included in this article.

## Author contributions

CY: Conceptualization, Data curation, Formal analysis, Software, Writing – original draft. RZ: Data curation, Methodology, Writing – original draft. YZ: Formal analysis, Methodology, Validation, Writing – original draft. YL: Methodology, Resources, Supervision, Writing – review & editing. TW: Conceptualization, Formal analysis, Writing – review & editing. RZ: Data curation, Software, Writing – review & editing. ML: Supervision, Visualization, Writing – review & editing. JZ: Funding acquisition, Supervision, Visualization, Writing – review & editing.
